# Genome-wide analysis of gene expression and protein secretion of *Babesia canis* during virulent infection identifies potential pathogenicity factors

**DOI:** 10.1038/s41598-017-03445-x

**Published:** 2017-06-13

**Authors:** Ramon M. Eichenberger, Chandra Ramakrishnan, Giancarlo Russo, Peter Deplazes, Adrian B. Hehl

**Affiliations:** 1 Institute of Parasitology, University of Zurich, Zurich, Switzerland; 2 Functional Genomics Center Zurich, Zurich, Switzerland

## Abstract

Infections of dogs with virulent strains of *Babesia canis* are characterized by rapid onset and high mortality, comparable to complicated human malaria. As in other apicomplexan parasites, most *Babesia* virulence factors responsible for survival and pathogenicity are secreted to the host cell surface and beyond where they remodel and biochemically modify the infected cell interacting with host proteins in a very specific manner. Here, we investigated factors secreted by *B*. *canis* during acute infections in dogs and report on *in silico* predictions and experimental analysis of the parasite’s exportome. As a backdrop, we generated a fully annotated *B*. *canis* genome sequence of a virulent Hungarian field isolate (strain BcH-CHIPZ) underpinned by extensive genome-wide RNA-seq analysis. We find evidence for conserved factors in apicomplexan hemoparasites involved in immune-evasion (e.g. VESA-protein family), proteins secreted across the iRBC membrane into the host bloodstream (e.g. SA- and Bc28 protein families), potential moonlighting proteins (e.g. profilin and histones), and uncharacterized antigens present during acute crisis in dogs. The combined data provides a first predicted and partially validated set of potential virulence factors exported during fatal infections, which can be exploited for urgently needed innovative intervention strategies aimed at facilitating diagnosis and management of canine babesiosis.

## Introduction

Apicomplexan parasites have evolved a plethora of targeted interactions with their host organisms and cellular machinery to elicit modification of gene expression and cellular functions. These modifications are critical for invasion and for establishment of intracellular niches for parasite replication, differentiation, and persistence, by securing nutrient import and mediating immune evasion^1^. As a result, host-parasite relationships in intracellular Apicomplexa can be viewed as co-adaptations driven by co-evolution of parasites with their hosts^2^. Most importantly, this complex interplay is directly linked to pathogenicity and virulence which characterize symptomatic infections with these protozoan parasites^3–8^.

Three genera of this phylum represent a triad of phylogenetically related hemoprotozoa: *Plasmodium* (human and animal malaria), *Theileria* (theileriosis in livestock), and *Babesia* (severe disease and death in cattle, horses, dogs, with some zoonotic potential). They are the causative agents of important human and/or animal diseases with high morbidity and mortality, responsible for significant economic burdens and healthcare challenges worldwide^9^. The clinically relevant part of the complex, heteroxenous *Babesia* life cycle takes place during asexual proliferation of parasites in red blood cells (RBC) of the mammalian intermediate host. This represents a distinct specialization of *Babesia* for growth in RBC, i.e. cells which lack organelles, as well as endocytic and exocytic membrane transport machinery^10, 11^.

Canine babesiosis is an emerging tick-borne disease of dogs with global distribution caused by morphologically distinctive *Babesia* species^12–14^. The geographical distribution of the disease caused by *B*. *canis*, the species responsible for a virulent form of canine babesiosis in Europe and Western Asia, appears to extend, as demonstrated by an increasing number of cases in areas outside of the established endemic regions^15^. The clinical spectrum of *B*. *canis* babesiosis is broad, ranging from apparently silent, mild disease to fulminant and often fatal presentations^14^. Indeed, in many respects canine babesiosis is highly comparable to clinical human malaria^16, 17^.

Analysis of correlated genomic, transcriptomic and proteomic data lays out the molecular underpinning for investigating the role of exported factors in disease manifestation and progression. However, experimental investigation of *B*. *canis* biology is limited by the current lack of cultivation techniques for propagation of this parasite *in vitro*. Here, we implemented short term *in vitro* cultivation of infected RBC in combination with whole genome sequencing, annotation, and comparative genomic characterization of a virulent Hungarian *B*. *canis* field isolate. By correlating RNA-seq and protein mass spectrometry data, we provide the first account of factors exported by *B*. *canis* blood stages with potential links to host-pathogen interaction and acute virulence.

## Results

### Genome sequence of a virulent *Babesia canis* strain

**Figure 1:**
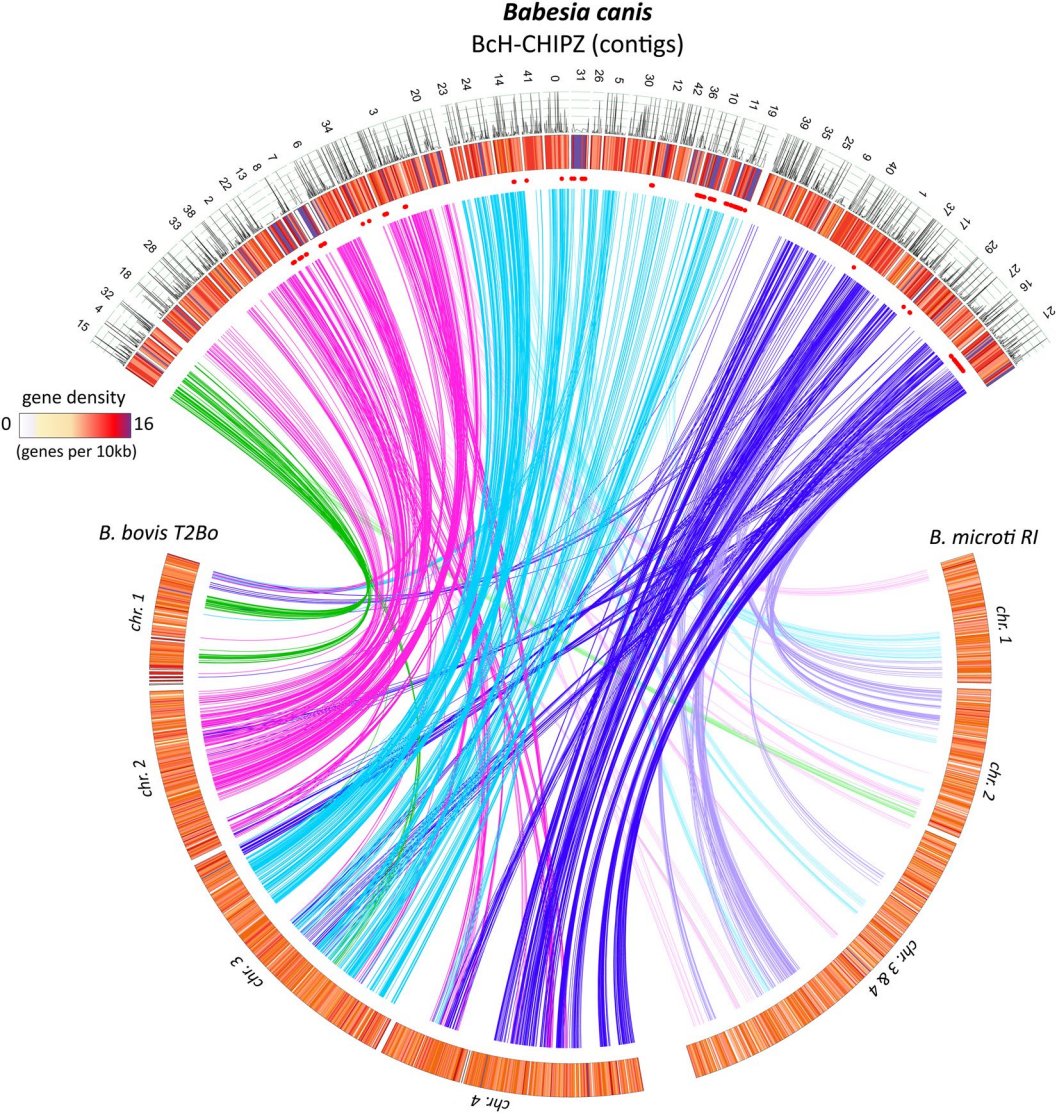
Genome characteristics of *Babesia* species. Assembled *B*. *canis* contigs (top arc) were arranged based on synteny to related *Babesia* species. The predicted arrangement of *B*. *canis* contigs on four chromosomes is indicated by colours of the lines linking conserved genes. Blood stage mRNA expression data for *B*. *canis* genes is included (line plot); gene density is colour coded and represented as number of genes per 10 kb (heatmap). Chromosomal distribution of *B*. *canis* VESA gene models is indicated with red dots.

Until recently, only a few genes from canine *Babesia* species have been characterized and global genomic data were not available. To provide the molecular underpinning for targeted approaches to diagnosis, vaccination and clinical management of canine babesiosis we undertook complete sequencing, assembly, annotation and characterization of the genome of a virulent *B*. *canis* strain, designated BcH-CHIPZ. The BcH-CHIPZ isolate originated from the blood of a Swiss dog that was infected, presumably through the bite of an infected tick, during a trip to Hungary. The isolate was characterized by genotyping and identified as double B-type by restriction fragment length polymorphism (RFLP) analysis in the Bc28-multigene family and the 18S rRNA gene^18^. We implemented a next-generation high throughput approach (PacBio® SMRT) for sequencing BcH-CHIPZ genomic DNA prepared after enrichment of infected RBCs (iRBCs). Assembly of the parasite-specific sequence reads after elimination of all reads from host DNA and gap-filling yielded 43 high-quality scaffolds at 55-fold average coverage representing a total of 7′002′180 bases with a median weighted contig size (N50) of 185 kb. Hence, the size of the *B*. *canis* genome lies between the phylogenetically related *B*. *microti* (6.5 Mb) and the *B*. *bovis* (8.2 Mb) genomes^19, 20^. The main features of the annotated *B*. *canis* genome and a comparison with other apicomplexan genomes are presented in Table 1. The quality of this assembly, based on the presence of core eukaryotic genes^21, 22^, indicates the *B*. *canis* genome sequence to be 92.6% complete as compared with the genome of the *T*. *gondii* ME49 type II reference strain^23^ and consistent with values of other *Babesia* genome sequences, which lie between 91.7% and 95.6% (Supplementary Document S1). A robust annotation of species-specific gene models was based on the analysis of orthologues as well as on synteny in related species (described below). Furthermore, whole genome mapping approach allowed contiguous alignment of the *B*. *canis* scaffolds with the 4 *B*. *bovis* chromosomes. This indicated a high degree of synteny with *B*. *bovis* chromosomes (Fig. 1).

**Table 1:** Genome characteristics of related apicomplexan hemoprotozoa (more information is provided in Supplementary Document S2).

Features	Species				
	*B*. *canis* [BcH-CHIPZ]	*B*. *bovis* [T2Bo]	*B*. *microti* [RI]	*Th*. *annulata* [Ankara]	*P*. *falciparum* [3D7]
Genome size (Mb)	7.0	8.2	6.5	8.4	23.3
# of chromosomes	4	4	4	4	14
# of scaffolds	43	12	3	7	14
G + C content (%)	45.8	41.5	36	32.5	19.4
Protein coding genes	3467	3706	3513	4082	5383
Mean gene size (bp)^1^	1044	1503	1327	1602	2292
% of coding regions	51.7	68	73	73	53
Gene density^2^	2020	2194	1816	2199	4374

^1^Excluding introns and UTRs; ^2^Genome size/number of protein coding genes.

### Genome-wide comparison of *B*. *canis* gene models with those of other Apicomplexa reveals species-specific genes

**Figure 2:**
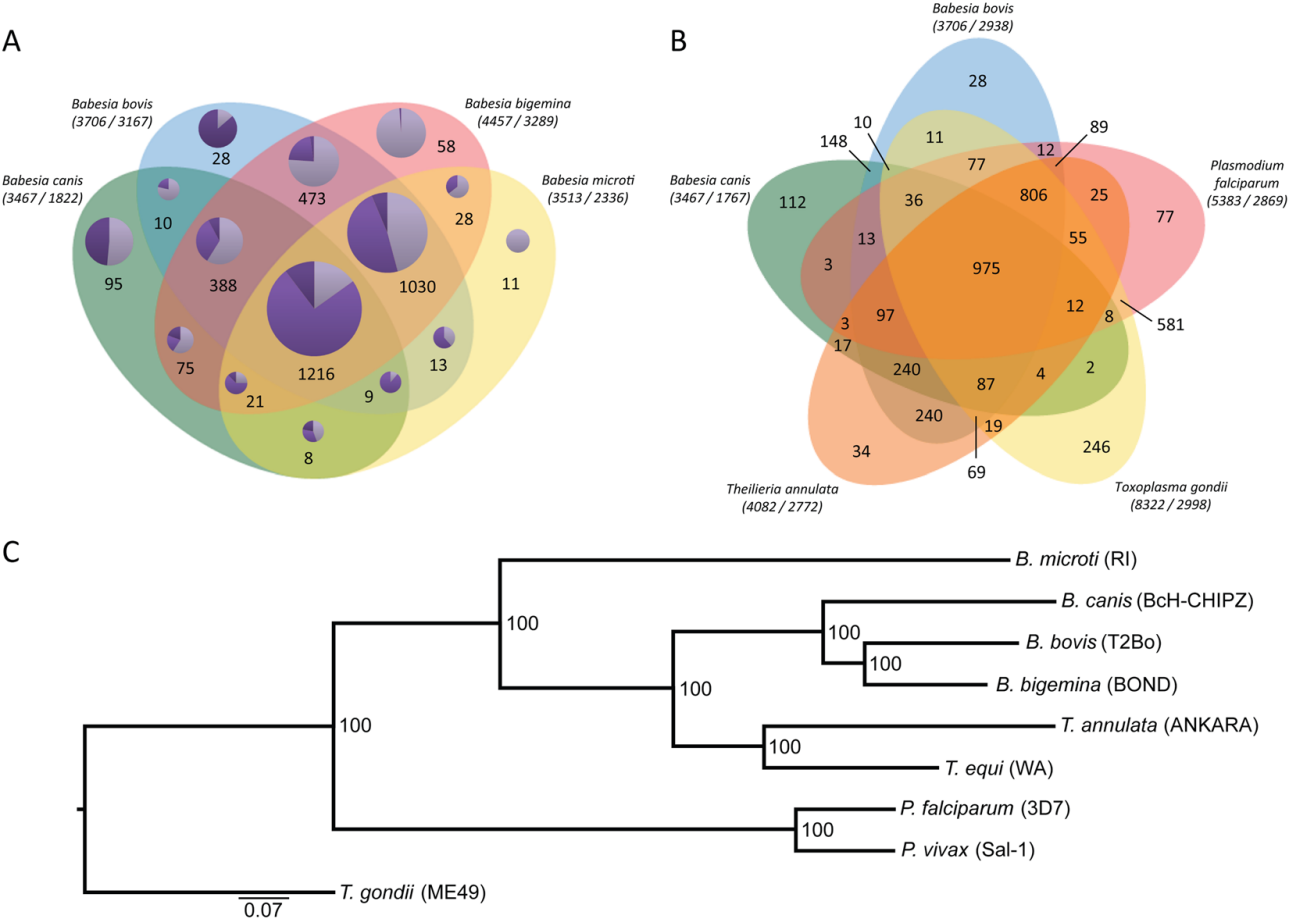
Comparative analyses of the *Babesia canis* BcH-CHIPZ genome. (**A**) Comparison of orthologous clusters in four *Babesia* species and (**B**) representative Apicomplexa species. The number of orthologous groups is indicated in the intersections. The total number of gene-models and clusters for each species is shown in brackets. Orthologous gene clusters in (**A**) are divided into three categories (pie chart colour code:  multigene families;  genes with annotations;  hypothetical genes). (**C**) Maximum likelihood phylogeny showing evolutionary relationship between selected *Babesia*, *Theileria* and *Plasmodium* species based on 975 shared orthologues in Apicomplexan species. The scale is in substitutions per site.

The *B*. *canis* gene models were grouped into orthologues in four representative apicomplexan parasites *B*. *bovis*, *Th*. *annulata*, *P*. *falciparum*, and *T*. *gondii* and the fully sequenced *Babesia* species *B*. *bovis*, *B*. *bigemina*, and *B*. *microti* using a Markov Cluster algorithm in OrthoMCL (Fig. 2A,B). The four *Babesia* species analysed here share a core set of 1216 pan-*Babesia* conserved orthologous groups (COG) (Supplementary Data S1). However, bovine and rodent *Babesia* genomes also share 1030 COGs which are not represented in the *B*. *canis* genome (Supplementary Data S2). The former and the pan-*Babesia* COGs together contain a high percentage of clusters with hypothetical proteins (46.9% and 38.1%, respectively), which is not surprising given the overall high proportions of proteins annotated as “hypothetical” (61.8% in *B*. *bigemina*, 49.9% in *B*. *bovis*, and 50.2% in *B*. *canis*). However, there was no specific enrichment with respect to protein domains and functions between the two groups. Functional classification of the 95 clusters unique to *B*. *canis* highlights potential species-specific innovations comprised of proteins annotated as “hypothetical” proteins (55.8%), surface proteins (proteins with VESA1-domains and merozoite surface antigen; 26.4%), a number of specified secreted proteins (9%), and various other proteins (8.6%) (Supplementary data S3). Analysis of the bovine *Babesia* shared orthologues revealed hypothetical proteins in 76.2% of the clusters analysed and predicted membrane proteins in 8.2% of the gene models. *Prima facie*, we interpret the diverged set of annotated genes in the different *Babesia* species as a reflection of the narrow and highly specific host range and a high degree of specialization for their respective vertebrate- and arthropod niches. This is based on the assumption that the diversity of encoded proteins is inversely correlated with the degree of specialization for different environments, niches, and lifestyles of a parasite, thus reflecting lineage-specific adaptations^24, 25^. Nevertheless, with 347 genes/Mb, the *B*. *canis* genome has a similar gene density as the *B*. *bovis* (372 genes/Mb) and the *B*. *microti* (354 genes/Mb) genomes, albeit some regions appear to have higher density than others (Fig. 1).

Despite significant species-specific features, analysis of orthologues revealed a considerable degree of synteny between the genomes of *B*. *canis* and *B*. *bovis* compared with the genome of the rodent *B*. *microti* (Fig. 1). This is also reflected in the phylogenetic analysis based on a core set of 975 shared genes in representative apicomplexan parasites (Fig. 2B,C).

### Prediction of the *B*. *canis* exportome and analysis of mRNA expression levels

Exported proteins of apicomplexan parasites include virulence factors, *i*.*e*. parasite-specific proteins which interfere directly with host cell functions. As mature RBC lack *de novo* protein synthesis or trafficking pathways, membrane transport machinery for targeting these proteins via the cytoplasm to the iRBC surface and beyond are provided by the parasite. These parasite-induced cytoplasmic alterations of iRBC are critical for its development and directly linked to the severity of babesiosis^26^. As a first step to characterize parasite-secreted factors and surface proteins, we generated a predicted *B*. *canis* exportome based on the annotated BcH-CHIPZ genome. We used data mining tools to search for genes coding for predicted parasite surface- or iRBC-targeted factors. The criteria and prioritization for this selection were based on the presence of a canonical hydrophobic N-terminal signal peptide (cSP) sequence, an alternative non-classical secretion pathway (a/ncSP) signal, predicted transmembrane (TM) domain(s), a GPI anchor signal, or previously described domains of conserved apicomplexan secretory proteins and protein families (Supplementary Fig. S1; Supplementary Data S4). By this approach we generated a curated and parsed list of 509 genes coding for proteins that are predicted to be exported to the parasite surface and/or to the host, which corresponds to 14.6% of all *B*. *canis* gene models. This proportion is very similar to the 528 gene models (14%) representing the predicted *B*. *bovis* secretome^27^.

**Figure 3:**
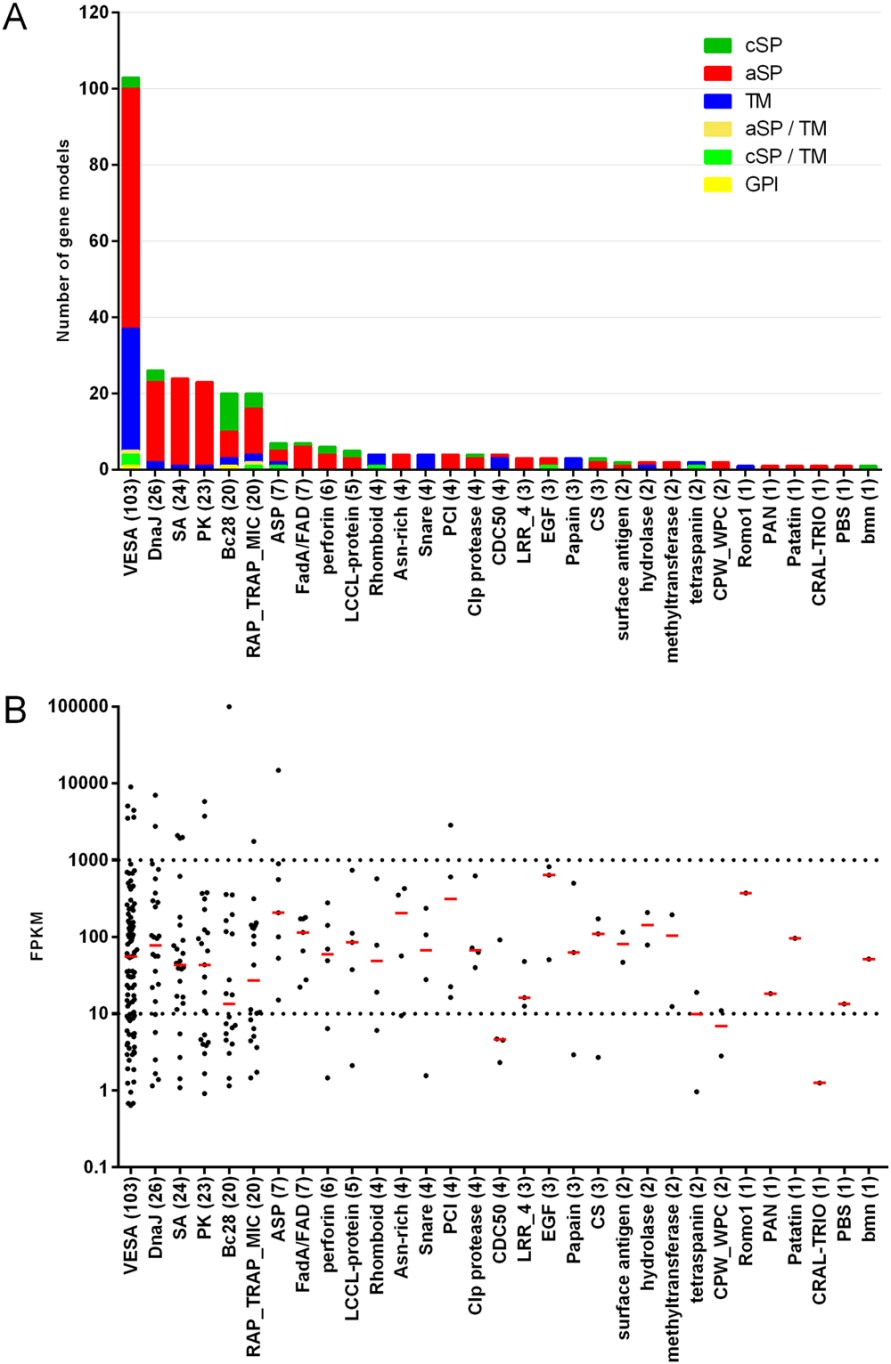
Exported protein families in *Babesia canis*. (**A**) *In silico* predicted exported protein families. Bar colours indicate the predicted route of secretion and/or type of membrane anchoring. cSP: canonical signal peptide; aSP: alternative (non-classical) secretory pathway; TM: transmembrane domain; GPI: predicted glycophosphatidylinositol anchor. (**B**) Transcript abundances of exported proteins. Each dot corresponds to the transcript abundance (FPKM) for each gene in the family during acute crisis in a splenectomised dog. Red bars indicate the median FPKM values in the presented protein family. FPKM: Fragments Per Kilobase of exon model per Million mapped reads.

All Apicomplexa express members of gene families that are exported to the surface of invasive stages or the plasma membrane of iRBC where they interact with the host immune system^28^. Members of the *Babesia* variant erythrocyte surface antigen (VESA) family on the surface of iRBCs^29^ comprise the largest protein family in *B*. *canis* (103 gene models) in the predicted exportome (Fig. 3A). Most VESA genes cluster together on few contigs, unlike other gene families of the predicted exportome which are distributed more randomly (Fig. 1). Furthermore, VESA genes are in regions of the genome with high gene density, suggesting frequent gene duplication events consistent with a driving selection pressure on these proteins associated with immune-evasion. *B*. *canis* merozoite surface antigens (MSA; 2 gene models in *B*. *canis*) as well as Bc28 family members (20 gene models) are highly abundant on the merozoite surface but are also shed and interact with the iRBC membrane and the host’s immune system^30, 31^. Currently, two members of the Bc28 gene family are characterized as major merozoite surface antigens playing a critical function in the interaction of merozoites with RBC^30, 32^. The second-largest family of exported proteins comprises secreted DnaJ (Hsp40) chaperones (26 members). *P*. *falciparum* DnaJ proteins have demonstrated roles in remodelling of iRBCs as well as in pathogenesis^33, 34^. The so-called “secreted antigens” (SA-1 and SA-3, 24 members) comprise yet another family of genes coding for exported proteins. Some SA genes have been identified in other *Babesia* species^35–38^. pBLAST analysis showed that the *B*. *canis* SAs are highly homologous (21 of 24 proteins with e-values < 10E-5) to those of *B*. *gibsoni*, which also propagates in dogs, and to a lesser extent to SAs of *B*. *bigemina* (3 of 24 proteins with e-values < 10E-5), which infects bovines. This supports the idea that host-specific factors drive expansion and diversification of these orthologous gene families. Thus, this gene family appears to be expanded in *Babesia* species infecting canines. In contrast, genes coding for smORF and spherical body proteins (SBP) appear to be specific to *B*. *bovis* and are without orthologues in the *B*. *cani*s genome^27, 39^. Accordingly, these protein families are also only represented in the bovine specific COGs. Proteins of the “secreted antigen family”, as the name indicates, are secreted beyond the iRBC and circulate in the blood of infected dogs but are otherwise not well characterized^31^. The predicted *B*. *canis* exportome also includes a group of 23 protein kinases. Secreted protein kinases and pseudokinases are known virulence factors in toxoplasmosis^40–43^. Kinases are also secreted into iRBCs in *Plasmodium*, where they are responsible *inter alia* for remodelling the host cell^44, 45^. However, functional data related to secreted kinases in *Babesia* are not available. Nevertheless, kinase inhibitors have been identified as potential novel drugs for the treatment of babesiosis in cattle^46, 47^.

To refine the predicted host-targeted *B*. *canis* exportome dataset we analysed the transcript levels of all 509 selected gene models in parasites harvested from a splenectomised dog. Evidence from a genome-wide RNAseq analysis showed transcription of 71.5% of the 509 exportome gene models (FPKM > 10; n = 364) (Fig. 3B). Remarkably, only a few members each of the five most abundant exported protein family genes (VESA-, DnaJ-, SA-, PK-, and Bc28- multigene families) with potential roles in antigenic variation and immune evasion were expressed at high levels (FPKM > 1000). The particular expression profile of the five gene families in this snapshot might be due to selection of a few specific phenotypes in the population at the peak of pathogenicity, but it should also be considered that the collected blood stage parasites do not represent a synchronously developing population. Nevertheless, this finding merits further investigation, in particular in the light of previous observations indicating that pathogenicity was associated with expression of a specific subset of VESA- and other exported genes in *B*. *bovis* infection^26^.

Further *in silico* characterization of the predicted *B*. *canis* exportome revealed a significant number of proteins (210/509) lacking a canonical hydrophobic signal sequence for co-translational insertion into the lumen of the endoplasmic reticulum, which is suggestive of non-classical secretion. This export pathway is generally known as ‘leaderless protein secretion’^48, 49^ and was described recently also in *P*. *falciparum* ^50–52^. We screened the *B*. *canis* exportome for additional motifs associated with protein trafficking into the host cell and beyond. First, we searched for matches to *Plasmodium* PEXEL export elements (RxLxE/Q/D) for trafficking beyond the parasitophorous vacuolar membrane (PVM)^53, 54^, which have been also identified in *T*. *gondii* and *C*. *parvum* ^27, 55^. Although *Babesia* builds a PVM during invasion of RBC it disappears within minutes and the parasite resides directly in the host cell cytoplasm^56^. A comprehensive comparative *in silico* analysis of protein export across 10 Apicomplexa species revealed PEXEL-like motifs (PLM) in *B*. *bovis* (but not in *T*. *parva*) with a role in retention of these proteins in so called spherical bodies and release in a cell-cycle dependent manner^27^. We found hits for the *B*. *bovis* PEXEL-like motifs within the first 100 N-terminal amino acids in 64.3% of cSP-containing proteins and only in 7.6% of predicted alternatively secreted *B*. *canis* proteins (Supplementary Data S4). Using a hidden Markov model to identify alternative motifs by *de novo* pattern discovery did not yield any additional hits.

### Expression levels of stage-specific genes identifies asexual and sexual *Babesia* blood stage parasites at acute crisis

**Figure 4:**
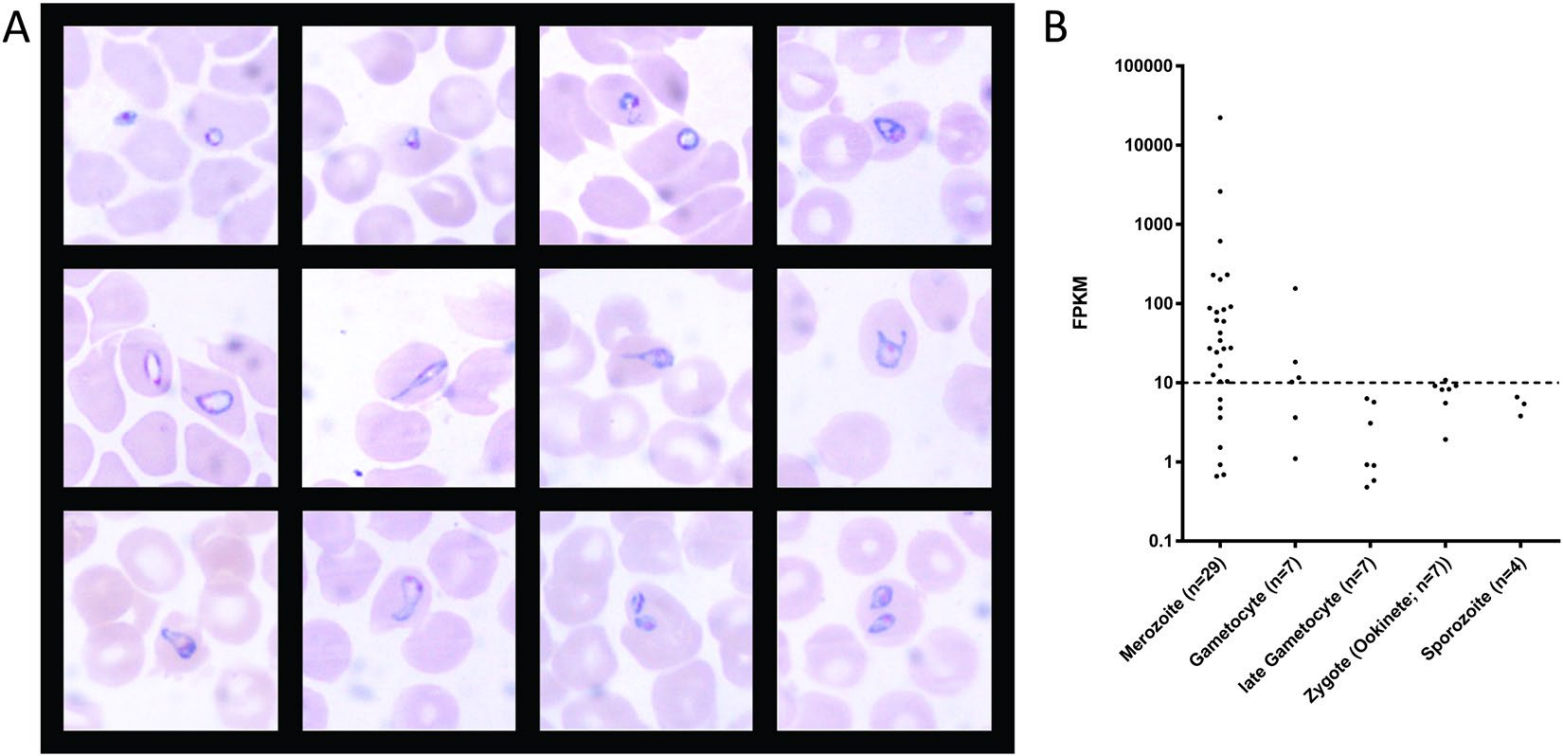
Specification of *B*. *canis* blood stages. (**A**) Representative Giemsa-stained blood smears from an experimentally infected dog with severe shock-like clinical signs showing typical pleiomorphic morphological appearance of *B*. *canis* parasites (parasitemia of 1.5% infected erythrocytes). (**B**) Transcriptome analysis of *B*. *canis* homologs of validated *Plasmodium* spp. stage-specific genes in a blood sample collected from an experimentally infected splenectomised dog with clinical signs consistent with acute canine babesiosis.

We collected biological material from three experimentally infected dogs, which all showed very similar rapid disease progression consistent with fatal babesiosis (described in ref. 57). The first mild clinical signs (e.g. lethargy) preceded acute crisis with early symptoms of septic shock by a maximum of 27 hours (Supplementary Fig. S3). In spite of the fulminant disease manifestation, a hallmark of canine babesiosis is a remarkably low parasitemia^12, 58, 59^ with typically <2% infected erythrocytes observed in the peripheral blood. The asexual erythrocytic stage of the parasite is entirely responsible for the morbidity and mortality associated with canine babesiosis. Morphological characterization of blood stage parasites revealed pleiomorphic forms in Giemsa-stained blood smears derived from a critically ill dog (Fig. 4A). Small and large ring stages were the most frequently detected forms, while only few pear-shaped forms could be identified. To characterize the parasite stages in iRBCs from experimentally infected animals on a molecular level in more detail, we used the high-throughput RNA-seq data to identify orthologous gene models with stage-specific expression in *Plasmodium* spp. We intersected the list of *B*. *canis* gene models with evidence for expression with a list of validated *Plasmodium spp*. stage-specifically expressed genes (e-value threshold of 10E-3) (Supplementary Data S5). We found evidence for RNA-seq reads (threshold FPKM > 10) mapping to 22 of 29 conserved merozoite, and 4 of 7 early gametocyte genes in a blood sample collected from a splenectomised dog (Fig. 4B). The higher mRNA abundance of merozoite- (proliferative stage) compared with early gametocyte-specific orthologues (sexual stage) during severe disease was consistent with the microscopy data and suggested only marginal numbers of circulating sexual stages in this peripheral blood sample. Nevertheless, some evidence for early gametocyte stages was found, which could indicate that acute clinical crisis in infected dogs might trigger development of sexual stages. For example in *Plasmodium*, gametocyte formation occurs in bone marrow and aggravates clinical symptoms and disease outcome^60, 61^. A potential bone marrow tropism could explain many typical clinical signs in canine babesiosis, e.g. leukopenia and thrombocytopenia, associated with poor outcome in acute infections^57^. *B*. *canis* orthologues of the 18 identified invertebrate stage-specific proteins (flagellar “late” gametocyte-, ookinete- and sporozoite genes) described for *Plasmodium* had FPKM values between 0 and 10.8, indicating that no or only insignificant amounts of these mRNAs were present in the sampled parasite population. Based on the degree of orthologue conservation and the microscopy data we interpret this as paucity of late gametocyte stages in the peripheral blood (Supplementary Data S5).

### Shotgun proteomics and transcriptomics identify potential *B*. *canis*-specific secreted virulence factors during severe disease

Proteins involved in parasite-host interactions at acute crisis were identified by a comparative shotgun approach from *B*. *canis* infected and healthy host blood samples. Mass spectrometry datasets were generated from secreted soluble factors collected from pooled short-term cultures from three experimentally infected dogs at acute crisis and RBC cultures derived from healthy blood donors, as well as from RBC membrane fractions from experimentally infected animals and non-infected controls. We identified a set of abundant parasite-specific proteins in these fractions after filtering the data with the non-infected control sets. Based on their abundance in the iRBC and/or culture supernatant the identified secreted membrane-bound and soluble parasite proteins were considered candidate factors involved in host-pathogen interaction (Table 2; Supplementary Fig. S2).

**Table 2:** List *Babesia canis* specific candidates from culture supernatant derived and membrane-bound fractions.

Identification	GO-description^1^	SP	TM	GPI	AS	Apicomplexan orthologue^2^	Transcriptome evidence (FPKM)
*Specific* B. canis *secreted soluble proteins*:							
BcH000240	Hypothetical protein, conserved	1	6	0	0		no (0.7)
BcH000493	Hypothetical protein [VESA]	0	0	0	1	VESA (*B*. *bovis* BBOVII004140)	yes (673.5)
BcH001113	Hypothetical protein, conserved	0	0	0	0		yes (544)
BcH002928	hypothetical protein, conserved	1	0	0	0		yes (64.2)
BcH003445	Secreted antigen 3	0	0	0	1	Secreted antigen 3 (*B*. *gibsoni* BAH70325)	yes (26.4)
BcH000300	Merozoite surface protein (Bc 28.2)	1	0	0	0	*B*. *canis* specific	no (4.5)
BcH002050	SET domain-containing protein	0	0	0	0	SET- and MYND domain containing protein, putative (*B*. *bigemina* BBBOND0302680)	yes (147.6)
BcH002986	hypothetical protein	0	0	0	0		yes (60.1)
BcH003048	Profilin	0	0	0	0	Polypeptide of profiling (*B*. *bigemina* BBBOND_0104160)	yes (100.6)
BcH002141	Snare protein	0	1	0	0	Snare protein, putative (*B*. *bigemina* BBOND0107960)	yes (236.6)
BcH000974	Hypothetical protein, conserved	1	0	0	0	VESA (*B*. *bovis* BBOV_III006920)	no (4.1)
BcH000821	Spectrin repeat superfamily extracellular matrix binding	0	1	0	0	VESA (*B*. *bovis* BBOV_II004140)	yes (123.2)
BcH000175	Hypothetical protein, conserved	0	0	0	0		yes (57.9)
BcH002958	Hypothetical protein [membrane protein]	0	0	0	1	Membrane protein, putative (*B*. *bigemina* BBBOND_0105580)	yes (56.4)
*Specific* B. canis *host*-*RBC membrane presented proteins*:							
BcH000821	Spectrin repeat superfamily extracellular matrix binding	0	1	0	0	VESA (*B*. *bovis* BBOV_II004140)	yes (123.2)
BcH000300	Merozoite surface protein (Bc 28.2)	1	0	0	0	*B*. *canis* specific	no (4.5)
BcH000299	Merozoite surface protein (Bc 28.1)	1	1	1	0	*B*. *canis* specific	yes (18.3)
BcH002699	Histone h2a	0	0	0	0	41-2-protein antigen precursor, putative (*B*. *bigemina* BBBOND_0103700)	yes (31.9)
BcH001932	Hypothetical protein, conserved	0	0	0	0		(yes 12.5)
BcH002268	Hypothetical protein, conserved	0	0	0	0		yes (111.9)
BcH001655	VESA	0	0	0	1	VESA (*B*. *bovis* BBOV_IV002850)	yes (884.1)
BcH001448	Hypothetical protein, conserved	0	0	0	0		no (0.9)
BcH003376	Secreted antigen 1	0	1	1	1	Secreted antigen 1(*B*. *gibsoni* ALE14558)	yes (142.5)
BcH000391	Hypothetical protein, conserved	1	0	0	0		yes (331.2)
BcH000895	Hypothetical protein, conserved	0	0	0	0		yes (26.4)

GO: gene ontology; SP: signal peptide; TM: transmembrane domain; GPI: AS: alternative secretion: GPI: glycophosphatidylinositol: VESA: variant erythrocyte surface antigen.

^1^Automated annotation by Blast2GO; [if no Blast2GO annotation could be assigned, best pBlast hit was provided in brackets].

^2^Other than hypothetical.

Not surprisingly, considering the limited understanding of apicomplexan pathogenicity determinants, the resulting *B*. *canis* dataset of exported proteins comprises 47.8% (11/23) proteins annotated as “hypothetical”. In addition, the following hits to functionally annotated proteins were identified: factors on cell surface membranes (from the parasite or the iRBC) which include 2 variant erythrocyte surface antigens (VESA) located on the surface of iRBC, a *Babesia* spp. membrane protein (homologous to *B*. *bovis*, *B*. *microti*, *B*. *bigemina*, *Theileria annulata*, *T*. *equi*, *T*. *orientalis*, *T*. *parva*, and *Cytauxzoon felis*), a spectrin repeat superfamily extracellular matrix binding protein with homology to *B*. *bovis* VESA, and 2 merozoite surface antigens of the Bc28 gene family. These proteins where shown to be involved in survival and virulence of several hemoprotozoa species^10, 18, 19, 30, 62, 63^. The dataset comprises SA1 and SA3, belonging to the above described third-largest exported family of proteins (secreted antigen family) suggesting a direct role in host-parasite interaction. The *B*. *canis* dataset also contains profilin, a secreted virulence factor described in *P*. *falciparum* and *T*. *gondii* with important roles in the process of crossing biological barriers during host cell invasion and egress^64, 65^, a SET-domain containing protein with putative function in lateral gene transfer in apicomplexans^66^, and a SNARE protein mediating membrane fusion of secretory vesicles with the plasma membrane^67, 68^. Furthermore, a secreted histone protein (classified as DNA-binding protein) was detected. Histone proteins were described to have moonlighting functions outside of the nucleus in *P*. *falciparum*, specifically at the interface between parasite and erythrocyte cytoplasm, i.e. the parasitophorous vacuole membrane^69^.

All hits in the proteomic dataset for which GO descriptions or blast homologies could be assigned were further validated using transcriptome evidence (threshold FPKM value > 10). Only Bc28.2, a known secreted *B*. *canis*-specific protein^30^ with a measured FPKM of 4.5, fell below this threshold. However, the correlation between transcript- and protein abundance is not linear and there is a complex relationship leading to notable discrepancies due to a range of factors such as protein- and mRNA turnover^70^. Taken together with additional evidence from the literature, these proteins are good candidates for factors that are presented to the host on the parasite or iRBC surface, or released in to the bloodstream and potentially involved in parasite persistence and virulence.

## Discussion

Secreted soluble and membrane-targeted parasite proteins have important roles at the host-parasite interface and are frequently virulence and pathogenesis factors. Systematic investigation of these interactions in infections with potentially fatal blood-borne parasites, such as *Babesia canis*, which modify the host cell, its microenvironment, as well as host physiology in multiple ways, is a challenge. Moreover, these questions cannot be addressed with biological material from regular patients from an animal clinic and the parasite is difficult to cultivate. Hence, in this study, we used experimental infections of dogs in a controlled setting to gather data on disease progression, clinical parameters, and parasite development. We report the first genome and gene expression data of the apicomplexan *B*. *canis* with a focus on exported factors secreted during acute infection. Acute *B*. *canis* infection with fulminant disease progression is associated with high mortality and characterized by an excessive inflammatory response caused by protozoal sepsis. This is caused by dysregulation of pro- and anti-inflammatory mechanisms during the acute phase of the disease, resulting in shock and end-organ failure^71–73^ (Supplementary Fig. S3). Interestingly, fatal *B*. *canis* infections (natural and experimentally induced) are invariably associated with at most moderate non-haemolytic anaemia and very low parasitemia^12, 57, 59^. This strongly suggests that potent secreted parasite factors are responsible for the severe pathogenicity of canine babesiosis. Hence, these as yet uncharacterized, secreted virulence factors are prime targets for the development of vaccines but also diagnostic reagents as a prime goal in clinical research.

Several major secreted factors inducing acute pathogenicity or persistence of hemoprotozoan parasites have been described in *P*. *falciparum* and *B*. *bovis* ^74^. Following RBC invasion, *B*. *bovis* exports VESA proteins to the surface of the host cell modifying the iRBC membrane to induce cytoadherence of iRBC at capillary and postcapillary venous endothelia^75–77^. VESA expression is subject to rapid antigenic variation defined as the serial clonal replacement of the expressed VESA gene in daughter merozoites before invasion of a new RBC. This gives rise to antigenically distinct parasite populations, which complicates mounting of an effective host immune response. Antigenic variation, i.e. exclusive sequential expression of surface antigen family members is likely a sub-phylum-specific immune-evasion strategy in hematozoa documented in several *Babesia* and *Plasmodium* species including *B*. *rodhaini*, *B*. *microti*, *B*. *bigemina*, *P*. *falciparum* and *P*. *vivax* ^8, 78^. Not surprisingly, the largest exported protein family in *B*. *canis* consists of the variant erythrocyte surface antigen (VESA) proteins. Indeed, a recent study using comparative transcriptomics and proteomics of attenuated and virulent *B*. *bovis* strains showed a significantly increased diversity of upregulated VESA genes in virulent strains^26^. In the present study, we detected a high number (74/103) of transcribed VESA genes as well as their products in the *B*. *canis* secreted proteome. Hence, adherence of *B*. *canis* iRBC to endothelial cells is one explanation for the apparent discrepancy between the low parasitemia observed in peripheral blood samples and the severity of the disease, consistent with sequestration of parasites and removal from the peripheral circulation. Correspondingly, using parasites isolated from a peripheral blood sample for the transcriptomic and proteomic studies might introduce a bias with respect to the detection of important pathogenicity factors. Direct comparison of proteomic and transcriptomic data has to be interpreted with caution, since the RNA-seq data was generated from parasites isolated from a splenectomized animal. The peripheral blood of splenectomized individuals would present a dominance of a sub-population of mature asexual and immature sexual parasites in iRBCs that failed to sequester, because they are not expressing appropriate ligands for cytoadherence, as repeatedly demonstrated in human *P*. *falciparum* ^79, 80^. Modulation of the adhesive properties of iRBCs by the spleen has been discussed, although no underlying mechanism has been identified^81, 82^. For example, in splenectomized rhesus monkeys infected with *Plasmodium knowlesi* a vast reduction in the expression of SICAvar genes, corresponding to their immunovariant adhesins, was demonstrated^83^. In contrast, *P*. *vivax* which preferentially invades reticulocytes passage all asexual blood stages through the spleen and show spleen-specific cytoadherence important for the development of a chronic disease by encoding equivalent vir-genes^8^. Nevertheless, the responsible *Plasmodium* var/vir- and the *Babesia* ves genes differ in structure, sequence, and in their biochemical properties^74^. In contrast to in *P*. *falciparum* var genes, *B*. *bovis* appears to antigenically vary its surface by a combination of segmental gene conversion events from inactive ves genes into an actively transcribed locus with *in situ* gene switching (clonal activation of genes)^84^.

Shedding of extracellular (exosome-like) vesicles (EVs) as carriers for proteins, lipids and RNA for intra- and inter-species communication (reviewed in refs 85 and 86) was identified recently as a non-classical export pathway for a completely new class of important parasite-derived factors. In *P*. *falciparum* infections, the concentration of EVs in the blood is positively correlated with severe disease suggesting an important role in pathogenesis and immune modulation^87^. Strikingly, the *B*. *canis* BcH-CHIPZ genome encodes homologues of important structural proteins and enzymes frequently found in exosomes, including heat shock proteins (e.g. HSP70 and 90), tetraspanins and ALIX protein, TSG101/ESCRT protein, 14-3-3 protein, thioredoxin peroxidase, histones, and the RAB GTPases 5, 7, 11, as well as a RAB GDI^85, 88, 89^. Due to the mechanism for their biogenesis, exosomes often contain endosome-associated proteins, such as the SNARE proteins also detected in the *B*. *canis* culture supernatant. In *P*. *falciparum*, a SNARE protein was localized proximal to the plasma membrane where it is possibly involved in membrane trafficking events associated with the parasite’s food vacuole^68^. Hence, EVs in *B*. *canis* infections could be crucial vehicle carriers for the dissemination of pathogenicity factors that are linked to acute and severe disease, including coding and non-coding RNA which can interfere with host cell gene expression. In fact, secretion via EVs could account for many of the non-classically secreted proteins detected in the *B*. *canis* proteomic datasets.

We identified several proteins with moonlighting functions which have been identified as virulence factors in related Apicomplexa. For example profilin (pfn) was shown to have important roles in the process of crossing biological barriers during host cell invasion and egress, in addition to its classical role as a regulator of actin dynamics^65^. The structure of the *P*. *falciparum* pfn accommodates additional domains with as yet unknown functions, which are potentially involved in the parasite-host interplay^64^. Another interesting finding was a histone protein in the iRBC membrane fraction. In several *Plasmodium* species, a histone protein methylated at position L9 was detected in the PV membrane^69^. The conserved PVM localization in *Plasmodium* indicated that histones have evolved additional functions in invasion and interaction with the host. As *Babesia* are not contained within a PV, the potential moonlighting function of this protein will have to be further investigated. Furthermore, histones are commonly found in EVs^90^. We also detected a *B*. *canis* SET-domain containing protein (BcSET) in the secreted soluble fraction. SET proteins are involved in diverse mechanisms such as transcriptional regulation, enhancer function, mRNA splicing, DNA replication, and DNA damage response^91^. An exported SET exerting an immune-suppressive function by transcriptional silencing of host gene expression was described in the pathogen *Bacillus anthraxis* ^92^. Interestingly, deregulation of gene expression due to SET domain-containing proteins is correlated with unfavourable clinical outcomes in various forms of cancer^93^.

Members of the Bc28 and SA gene families are among the most prominent *B*. *canis* secreted factors, and have recently gained attention as candidates for innovative vaccine strategies and in diagnostics^30, 31^. Bc28 gene products are highly represented on the merozoite surface, but are also shed and can interact with the iRBC membrane^30^. Bc28 belong to a multigene family composed of polymorphic genes. Different *B*. *canis* strains in Europe are associated with different mortality rates and show genetic heterogeneity in Bc28.1, detectable by a PCR-RFLP test^18^. It has been suggested that this variable repertoire has evolved to allow the parasite to evade host immune responses, although merozoite surface proteins of *Babesia* spp. are thought to be the main targets of the adaptive host immune response^32, 94^. However, whether all members are directly involved in immune evasion and virulence, or even show stage-specific behaviour, will have to be investigated in more detail.

In the absence of reverse genetic techniques for *B*. *canis*, sequencing and annotation of a virulent *B*. *canis* strain together with the described parasite blood stage proteome and transcriptome provides a powerful underpinning for designing targeted intervention strategies such as the development of novel diagnostic tools, effective vaccines, and innovative drugs.

## Methods

### Parasites

Biological materials for the different experiments were collected from experimentally infected dogs. Animals were inoculated intravenously with 1 × 10^6^ parasitized erythrocytes from a cryo-conserved field isolate, derived from a naturally infected Bernese mountain dog from Switzerland that had been exposed during a trip to Hungary. All experimentation with animals and protocols was in complete compliance with the strict Swiss animal welfare standards and regulations and approved by the responsible authorities (Veterinary Office of the Canton of Zurich; permission number 122/2012) previous to the study, including round the clock surveillance of the animals by a veterinarian. Whole blood was treated with citrate-phosphate-dextrose-adenine (12.3% CPDA-1, v/v) for anticoagulation under sterile conditions and prepared immediately after euthanasia. Blood samples for genomic and transcriptomic studies were collected from an experimentally inoculated splenectomised dog (4 years old). Splenectomy increased the final amount of parasites in the peripheral blood to approximately 4% infected erythrocytes. The biological material for the proteome analysis was collected from three experimentally infected adult beagles with a parasitemia of 1–1.75% (Supplementary Fig. S3). All samples were collected at the first signs of acute crisis detected by any clinical signs of acute shock or central nervous depression.

### Genome sequencing, assembly, and annotation pipeline

Genomic DNA (gDNA) was prepared from purified and host leukocyte-depleted^95^ infected blood from an experimentally infected, splenectomised dog. High-throughput genome sequencing was performed on a PacBio Single Molecule Real Time (SMRT) sequencing platform^96^ run on 4 SMRT cells. 5 μg of input gDNA was used for library construction. Sequenced fragments were filtered to eliminate *Canis familiaris* reads and processed for assembly with PacBio read-alignment software BLASR^97^. The final draft of the genome was obtained using a two-step procedure. De novo assembly was initially performed using the HGAP2 algorithm^98^ and the outcome was further polished using the PacBio proprietary analysis package Quiver, a reference-guided consensus tool used as part of the SMRT analysis. The resulting draft was subjected to gap-closing and further scaffolding using PBJelly v14.1.15^99^. Genome annotation was performed using different sources of evidence-based prediction on the Maker2 pipeline^100^, on ab initio gene predictor algorithms^101^ with *P*. *falciparum* 3D7^102^, *B*. *bovis* T2Bo^19^, and *T*. *annulata* Ankara^103^ gene models as templates retrieved from EuPathDB release 26^104^, and on Trinity-assembled de novo transcriptome evidence^105^. Repetitive genomic elements were identified and masked from annotation with RepeatMasker using the full Repbase database^106^. Redundant transcripts potentially representing sequencing errors or genetic polymorphisms were clustered with the cd-hit software^107^. Non-overlapping sequences of the different evidence datasets were generated from the gff-files with BEDTools genome arithmetic software^108^. Functional annotation of the gene-predictions was achieved by gene ontology mapping through Blast2Go v3.1^109^ using an upper cut-off e-value of <0.0005.

Full methodological details are provided in the Supplementary Material.

### RNA preparation and RNA-seq data analysis

Total RNA was prepared from host leukocyte-depleted *B*. *canis* blood stages using the Zymo Direct-zol RNA MiniPrep kit (Zymo Research) including an on-column DNase I (QIAGEN) digest. Paired-end, stranded sequencing of the cDNA library was performed on 1 lane of an Illumina HiSeq 2000 sequencer (Illumina Inc.). Measurement of expression level was based on fragments per kilobase of exon model per million mapped reads (FPKM), normalized by the length of the gene. A gene was considered expressed if its FPKM was at least 10. Full methodological details are provided in the Supplementary Material.

### Summary of computational genome analysis

The identification of potential homologues of *B*. *canis* genes to selected apicomplexan parasites (*B*. *bovis* T2Bo^19^, *B*. *bigemina* BOND^39^, *B*. *microti* RI^20^, *T*. *annulata* Ankara^103^, *P*. *falciparum* 3D7^102^, and *T*. *gondii* ME49^23^) was carried out by protein BLAST (Blast + executables v2.2.31) of each individual protein sequence. A e-value < 0.001 cut-off was considered appropriate. For comparative genomics analysis of species-specific genes and clustering of orthologues, OrthoMCL (v2.0.9) was used^110, 111^. Synteny analysis was performed in MCScanX^112^ by the pairwise determination of syntenic regions between species based on the order of orthologues from the OrthoMCL output with default values. *B*. *canis* contigs were arranged according to the syntenic relationship to *B*. *bovis* with ‘orderchr’ execution in Circos utilities. The graphical representation was made with Circos^113^. Whole genome phylogeny was based on the 975 identified OrthoMCL-orthologues shared among Apicomplexan species. Each of these gene models in 4 *Babesia* sp. (*B*. *canis*, *B*. *bovis*, *B*. *bigemina*, and *B*. *microti*), 2 *Theileria* sp. (*T*. *annulata* and *T*. *equi*), 2 *Plasmodium* sp. (*P*. *falciparum* and *P*. *vivax*) and *Toxoplasma gondii* was aligned using MAFFT v7^114^. Highly variable sites were trimmed using trimal^115^. A supermatrix of the alignments was concatenated using FASconCAT^116^. Based on this alignment a maximum likelihood phylogenetic tree was constructed using RAxML^117, 118^ based on amino acid input, gamma model, LG substitution matrix and 100 initial bootstrap (bootstrap percentage support is shown along the branching nodes), whereas the tree was rooted using *T*. *gondii* as an outgroup. Prediction of the *B*. *canis* exportome was based on several in silico analysis tools, databases, and manual curation (Supplementary Fig. S1). Full methodological details are provided in the Supplementary Material.

### Proteomic studies

Proteomic analyses were based on secreted soluble *Babesia* antigens and membrane-bound *B*. *canis* proteins. Culture supernatant was collected after 18 h of short term *in vitro* parasite cultures of *B*. *canis* RBC in serum-free medium prepared from anticoagulated whole blood from experimentally inoculated dogs. Similarly, blood from a healthy canine blood donor was cultured and supernatant was processed as a control. Isolation of membrane bound proteins was based on anticoagulated, leucocyte-depleted blood. iRBC were concentrated by Percoll (GE Healthcare) gradient centrifugation^119^ and parasites were released from the iRBC by streptolysin-O mediated lysis^120^ and removed by an additional Percoll gradient centrifugation step^121^. Membrane proteins were collected by homogenization and repeated ultracentrifugation steps, mild sonication and acetone precipitation. The same protocol was used to collect proteins from uninfected erythrocyte membranes. All samples were collected and stored at −80 °C until further use.

Protein preparations were separated on one dimensional SDS-PAGE under sterile and reducing conditions. Each stained gel was cut into 8 equal sections and in-gel trypsin digestion was carried out for subsequent mass spectrometry analysis. Liquid chromatography-MS/MS was performed on a Q-exactive mass spectrometer (Thermo Scientific) equipped with a nanoAcquity UPLC (Waters Corporation).

Following peptide data acquisition, searches were performed using the MASCOT search program against a database built on the *B*. *canis* annotated genes with a concatenated decoy database supplemented with commonly observed contaminants and the Swissprot database to increase database size, or against NCBI database for *Canis lupus familiaris*. Identified hits were filtered in Scaffold Viewer V4 (Proteome Software, Portland, US) based on stringent parameters to reach false discovery rates of <5%. Furthermore, all datasets were corrected for obvious host- and environmental contaminants by an additional pBLAST search of the NCBI non-redundant protein sequences database with the single peptide hits.﻿

### Data Availability

The annotated B. canis genome will be made accessible via PiroplasmaDB hosted﻿ by EuPathDB (http://piroplasmadb.org/piro/).

## Electronic supplementary material

Supplementary information

Supplementary Data S1 to S6
