# Supplementary material for: Genome-wide analysis of gene expression and protein secretion of Babesia canis during virulent infection identifies potential pathogenicity factors
Source: Sci Rep. 2017 Jun 13;7:3357. doi: 10.1038/s41598-017-03445-x (PMC5469757; doi:10.1038/s41598-017-03445-x)

Genome-wide analysis of gene expression and protein secretion of *Babesia canis* during virulent infection identifies potential pathogenicity factors

Ramon M. Eichenberger[1], Chandra Ramakrishnan[1], Giancarlo Russo[2], Peter Deplazes[1], Adrian B. Hehl[1*]

[1] Institute of Parasitology, University of Zurich, Zurich, Switzerland

[2] Functional Genomics Center Zurich, Zurich, Switzerland

Supplementary information

Supplementary Document S1 2

Supplementary Document S2 3

Supplementary Methods 5

Isolation of genomic DNA, library preparation and genome sequencing 5

Genome assembly and annotation 6

Analysis of genome completeness 8

RNA preparation, wet lab transcriptomics, and transcriptomic data analysis 8

Functional annotation and characterization 9

Prediction of exported gene models 10

Samples for proteomic studies 11

Mass spectrometry and protein identification 12

References 14

Supplementary Figure S1 16

Supplementary Figure S2 17

Supplementary Figure S3 18

Supplementary Document S1. CEGMA completeness and completeness relative to *Toxoplasma gondii* core eukaryotic gene families in selected apicomplexan species.

[Species]	[CEGMA completeness]	[Completeness relative to *T. gondii* KOGs]
T. gondii Me49	84.7%	100%
E. tenella Houghton	83.1%	98.1%
P. falciparum 3D7	75.8%	-
B. canis BcH-CHIPZ	75.4%	92.6%
B. bovis T2Bo	70.2%	95.5%
B. bigemina BOND	71.8%	95.6%
B. microti RI	66.9%	91.7%

Supplementary Document S2. Genome overview

**Genome**	***B. canis* [BcH-CHIPZ]**	***B. microti* [RI]**	***B. bovis* [T2Bo]**	***B. bovis* [C9.1]**	***B. div.* [1802A]**	***B. div.* [Rouen1987]**	***B. bigem.* [BOND]**	***Th. equi* [WA]**	***Th. parva* [Muguga]**	***Th. Annu.* [Ankara]**	***P. falc.* [3D7]**
**Year**	2015	2012	2007	2014	2014	2014	2014	2012	2008	2008	2002
Origin	Hungary	USA	Texas (USA)	Mexico	France	France	Argentina	USA	East Africa (Kenia?)	Turkey?	
Host	Dog	Human	Cow	Cow	Cow	Human	Cow	Horse	Cow	Cow	Human
**Feature**											
Genome											
Size (Mbp)	7	6.5	8.2	7.61	9.58	8.97	13.8	11.6	8.3	8.4	23.3
Number of chromosomes		4	4				4	4	4	4	14
Number of scaffolds	43	3	12	46	81	482	6		8	7	14
G+C content (%) =pGC	45.8	36	41.5	42	42	46	51	39.5	34.1	32.5	19.4
Genes											
Number of genes	3467	3513	3706	3726	4134	4097	4457	5330	3796	4082	5383
Mean gene length (bp)	1044	1327	1503	1501	1487	1439	1531	1472	1407	1602	2292
Mean gene length including introns (bp)	1200	1471	1609						1654	1802	2590
Gene density (bp per gene)	2020	1816	2194	2154	2321		2306	2185	2059	2199	4374
Coding regions (%)	51.7	73	68	70.5	64.0		66.3	69	68	73	53
Coding regions including introns (%)	59.4	81	73						80	82	59
Number of genes with introns (%)	59	70	60	62.5	59	57.5	58.4	52.4	75	71	54

Exons											
Number per gene	2.6	3.3	2.8						2.7	3.9	2.6
Mean length (bp)	405	397	547						514	416	904
Total length (%)	51.7	73	68						68	73	53
Introns											
Number per gene	1.6	2.3	1.7						2.6	2.9	1.6
Number per gene presenting intron	2.7	3.4	2.9						3.5	4	2.9
Mean length (bp)	100	61	60						94	70	167
Total length (%)	8	8	5						12	9	6
Intergenic regions											
Mean length (bp)	820	346	585					550	405	398	1784
Total length (%)	40.6	19	27						20	18	41
RNAs											
Number of tRNA genes		44	70						71	47	72
Number of 5S rRNA genes		2	ND						1	3	3
Number of 5.8S/18S/28S r RNA units		2	5						8	1	13

Supplementary Methods

Isolation of genomic DNA, library preparation and genome sequencing

Whole blood from the experimental inoculated splenectomized dog was used. Washed blood samples were passed twice through a cellulose powder column (medium fibre powder; Sigma-Aldrich) to deplete host leukocytes (Rodriguez et al. 1986; Sriprawat et al. 2009). First fraction was used for RNA preparation (see below). After a three times repeated wash step with ice cold RPMI-1640 (R8758, Sigma-Aldrich, St. Louis, USA). DNA was extracted from the iRBC peletts using several columns of the QIAamp® DNA Mini kit (Qiagen) and the blood protocol according to the manufacturer's insturctions. Eluate from these columns was further purified and concentrated by AMPure XP beads (Beckman-Coulter) and library for high-throughput sequencing was prepared. All steps were performed in siliconized tubes and with siliconized tips.

High-throughput genome sequencing was based on PaciBio Single Molecule Real Time (SMRT) sequencing (Eid et al. 2009). The SMRT bell, was produced using the DNA Template Prep Kit 1.0 (Pacific Biosciences p/n 100-259-100). The input genomic DNA concentration was measured using a Qubit Fluorometer dsDNA Broad Range assay (Life Technologies p/n 32850). 5µg of gDNA were mechanically sheared to an average size distribution of 5-10Kb, using a Covaris gTube (Kbiosciences p/n 520079). A Bioanalyzer 2100 12K DNA Chip assay (Agilent p/n 5067-1508) was used to assess the fragment size distribution. 2 µg of sheared gDNA was DNA damage repaired and end-repaired using polishing enzymes. A blunt end ligation reaction followed by exonuclease treatment was performed to create the SMRT bell template. A Blue Pippin device (Sage Science) was used to size select the SMRT bell template and enrich the big fragments > 5Kbp. The sized selected library was quality inspected and quantified on the Agilent Bioanalyzer 12Kb DNA Chip and on a Qubit Fluorimeter (Life technologies) respectively.

A ready to sequence SMRT bell-Polymerase Complex was created using the P6 DNA/Polymerase binding kit 2.0 (Pacific Biosciences p/n 100-236-500) according to the manufacturer instructions. The Pacific Biosciences RS2 instrument was programmed to load and sequence the sample on 4 SMRT cells v3.0 (Pacific Biosciences p/n100-171-800), taking 1 movie of 240 minutes each per SMRT cell. A MagBead loading (PacBio p/n 100-133-600) method was chosen in order to improve the enrichment the longer fragments.

After the run, a sequencing report was generated for every cell via the SMRT portal, in order to assess the adapter dimer contamination, the sample loading efficiency, the obtained average read-length and the number of filtered sub-reads.

Genome assembly and annotation

Long fragments sequenced on the Pacific Bioscience RSII had the SMRTbell adapters removed and filtered according to a minimum read length of 50 nucleotides and a minimum read quality of 75%. Moreover, contamination from fragments coming from the host *Canis familiaris* were also excluded following a mapping step with PacBio read-alignment software BLASR (Chaisson and Tesler 2012). The final draft of the genome was obtained using a two-step procedure. De novo assembly was initially performed using the HGAP2 algorithm (Chin et al. 2013) and the outcome was further polished using Quiver, a reference guided consensus tools developed as part of the SMRT analysis, PacBio proprietary analysis package. The resulting draft was carried over for gap-closing and further scaffolding using PBJelly v14.1.15 (English et al. 2012). BLASR was provided with the following settings: minMatch = 8, sdpTupleSize = 8, minPctIdentity = 75, bestn = 1, nCandidates = 10, maxScore = 500, and noSplitSubreads was activated.

Genome annotation was performed using different sources of evidence. First evidence was based on the Maker2 pipeline (Stanke and Waack 2003). Augustus (Version 3.1) (Korf 2004)

was used as ab initio gene predictors using initially *P. falciparum* as model organism. The Trinity-assembled de novo transcriptome was used as transcript evidence (RNA seq is described below). Protein sequences from UniProt were used as homology-based evidence. Repetitive genomic elements were identified and masked from annotation with RepeatMasker (http://www.repeatmasker.org) using the full Repbase database (Jurka et al. 2005). The Maker2 annotation was run a first time using the de novo transcriptome directly to infer gene predictions (i.e. est2genome=1) and a second time incorporating the transcript-annotation file (in .gff format) obtained from the first run (i.e. “pass-through” aided annotation). De novo transcriptome assembly (RNA isolation and processing is described below) was performed using the Trinity software suite v.2013-8-14 (Grabherr et al. 2011) using default parameters. The likely coding DNA sequences (CDS) and corresponding proteins within the de novo transcriptome assembly were estimated with Transdecoder (http://transdecoder.sourceforge.net/). Redundant transcripts potentially representing sequencing errors or genetic polymorphisms were clustered with the cd-hit software (Fu et al. 2012). This first approach resulted in 2187 gene-predictions. In order to improve the dataset, a second evidence was generated by ab initio gene prediction. Augustus was trained with complete gene sets to perform ab initio annotation with *P. falciparum* 3D7, *B. bovis* T2Bo, and *T. annulata* Ankara as model organisms, retrieved from EuPathDB release 26 (Aurrecoechea et al. 2010). Non-overlapping sequences to the first evidence were generated from the gff-files with BEDTools genome arithmetic software (Quinlan 2014). This second approach resulted in additional 690 gene-predictions. Third dataset was based on Trinity-assembled de novo transcriptome transcript evidence and curation of the hits. The non-overlapping sequences to the first two sources of evidence resulted in additional 590 gene-predictions.

Analysis of genome completeness

We initially used CEGMA (Parra et al. 2007) to identify orthologues of core eukaryotic gene families (KOGs) within the *Babesia canis* genome assembly and compared this to values found in *Toxoplasma gondii* ME49, *B. bovis* T2Bo, *B. microti* RI, *Plasmodium falciparum* 3D7, and *Eimeria tenella* Houghton genomes. The values for all *Babesia* genomes were much lower than those for *Toxoplasma*, but consistent within *Babesia* and comparable to that of *P. falciparum* (Supplemental document S1). Hence, we concluded this might be due to genome fragmentation rather than genome incompleteness. Similar to the approach performed in *Eimeria* genomes (Reid et al. 2014), we then analysed the genes relating to each KOG found in *Toxoplasma* (Parra et al. 2009) but not in *B. canis* and quested for one-to-one orthologues for these using OrthoMCL. In 46 of 63 cases we identified the missing KOG member in the *B. canis* genome and adjusted the CEGMA completeness values by adding in the newly identified KOGs.

RNA preparation, wet lab transcriptomics, and transcriptomic data analysis

Host-contamination depleted *B. canis* blood stages were rapidly re-suspended in Trizol (Ambion/Life Technologies) and frozen at -80 °C. The RNA was then isolated using the Zymo Direct-zol RNA MiniPrep kit (Zymo Research) according to the manufacturer's instructions, including an on-column DNase I (QIAGEN) digest. The RNA quality was analysed using the Agilent RNA 6000 Pico Kit (Agilent) on a Bioanalyzer 2100 (Agilent) and quantified with a Qubit Fluorometer 1.0 (Life Technologies). Using the TruSeq RNA Sample Prep Kit v2 (Illumina Inc.), 0.1 – 1.0 µg of total RNA were enriched for mRNA by poly-A selection before double-stranded cDNA library generation, fragmentation, end-repair and poly-adenylation according to the manufacturer's protocol. TruSeq adapter were ligated and selectively enriched with PCR. The quality and quantity of the enriched library was validated using Qubit, the Caliper GX LabChip

GX (Caliper Life Sciences, Inc.) and quantitative PCR. The library was normalized to 10 nM in 10 mM Tris-HCl/0.1% Tween 20, pH 8. The TruSeq PE Cluster Kit v3-cBot-HS (Illumina Inc.) was used for cluster generation on cBot (Illumina Inc.) using 10 pM of normalized library. Paired end, stranded sequencing was performed on 1 lane using the Illumina HiSeq 2000 sequencer (Illumina Inc.) with 2 x 125 bp reads.

Measurement of expression level was based on Fragments Per Kilobase of exon model per Million mapped reads (FPKM) normalized by the unique length of the gene. Expression of a gene was assumed by a FPKM threshold of 10.

Functional annotation and characterization

Functional annotation of the gene-predictions was achieved by gene ontology mapping through Blast2Go v3.1 (Conesa et al. 2005) using a cut-off of E-Value < 0.0005. The identification of potential homologues of *B. canis* genes to selected apicomplexan parasites (*B. bovis* T2Bo, *B. bigemina* BOND, *B. microti* RI, *T. annulata* Ankara, *P. falciparum* 3D7, and *T. gondii* ME49) was carried out by protein blast (Blast+ executables v2.2.31) of each individual protein sequence. A cut-off of E-Value < 0.001 was considered as appropriate. Multiple alignments of protein sequences were performed using ClustalΩ (v2.1). For comparative genomics of species specific genes and clustering of orthologues, OrthoMCL (v2.0.9) was used (Li et al. 2003; Fischer et al. 2011), whereby pan-apicomplexan comparison included protein sets from *B. canis* CHIPZ, *B. bovis* T2Bo, *P. falciparum* 3D7, *T. gondii* ME49, and *T. annulata* Ankara, and pan-*Babesia* comparison includes protein sets from *B. canis* CHIPZ, *B. bovis* T2Bo, *B. bigemina* BOND, and *B. microti* RI, respectively. Available datasets were extracted from EuPathDB release 26 (Aurrecoechea et al. 2010).

Prediction of exported gene models

In a first stage, prediction of the *B. canis* exportome was based on different in-silico analysis tools, databases, and manual curation. Criteria for selection in the predicted exportome were based on the prediction of a canonical hydrophobic N-terminal signal peptide (cSP) by SignalP v3.0 (Bendtsen et al. 2004b) using conservative D-score discrimination of 0.46, prediction of transmembrane (TM) domain by TMHMM v2.0 (Krogh et al. 2001) using default settings, prediction of previously described and known apicomplexan secretory protein families using Markov-based method Pfam (Finn et al. 2014), functional gene ontology and pBlast data for protein homology detection to other apicomplexan (Pelle et al. 2015). Additionally, prediction of potential leaderless protein secretion was based by ab initio prediction of non-classical triggered protein secretion by SecretomP 1.0f using conservative NN-score cut-off of 0.6 (Bendtsen et al. 2004a). Prediction of GPI-anchors was performed using PredGPI server (Pierleoni et al. 2008) with the conservative model and an inclusion above 99% prediction probability. In silico GPI prediction without TM helices prediction were ignored and grouped according other evidences due to overestimation of GPI anchors (Pierleoni et al. 2008). Transmembrane domain-containing proteins were further analyzed for sub-cellular direction to the parasite (plasma) membrane based on ProtComp 9.0.

On a second stage, proteins witch could not be assigned to any known apicomplexan secretory protein family and with an explicit prediction of subcellular targeting were assigned to its subcellular direction (and eliminated from the list of potential host-targeting proteins). Apicoblast targeting was based on local ApicoAP v2.7.3 (Cilingir et al. 2012) based on SignalP 3.0 predicted signal peptides and *B. bovis* or *B. microti* as model organism. Mitochondrial targeting was predicted with MitPred v2.0 (Kumar et al. 2006) curated with TargetP v1.1 (Emanuelsson et al. 2000) prediction and pBLAST evidence. Nucleus targeting was based on

NucPred (Brameier et al. 2007). Ambiguous results were cross-validated with the ApiLocDB v3 whenever possible. Subcellular location was further validated by combination of TargetP and ProtComp 9.0 results. Finally, proteins which contain only a TM-domain and could not be assigned to a known host-targeting protein family were not considered to target the host, as these proteins are likely to be retained within the parasite. This approach resulted in a list of proteins targeting the parasite surface, iRBC membrane, and which are potentially secreted to the host cell cytoplasm or beyond.

Samples for proteomic studies

Secreted soluble B. canis *proteins*. Short term in-vitro cultures of *B. canis* were performed from anticoagulated whole blood (12.3% v/v citrate-phosphate-dextrose-adenine; CPDA-1) from experimental inoculated dogs and kept always at 37°C. After removing plasma, blood samples were washed and passed twice through a cellulose powder column (medium fibre powder; Sigma-Aldrich) to deplete host leukocytes. Subsequently, parasites were cultured for 18 hours in serum-free medium based on RPMI-1640 (Sigma-Aldrich), 25 mM HEPES (Sigma-Aldrich), 25 mM NaHCO3, 2 mM L-glutamine (Sigma-Aldrich) and 100 µg/ml neomycine (Sigma-Aldrich). Each culture consisted of a total volume of 10 ml with 5% packed cell volume (approx. 1% parasitaemia) in 90 mm petri dishes (Thermo Fisher) at 37 °C, 4% CO_2 and 3% O_2 cultured in a modular incubator chamber (MIC-101; Billups-Rottenberg). The medium containing the secreted soluble *Babesia* proteins was collected, centrifuged (2,000 x g for 10 min) and the supernatant concentrated with an Amicon filtration unit and PLGC (10,000 NMWL) membranes (both Merck Millipore, Darmstadt, Germany). Concentrated antigens from the two different media were stored at -80 °C until used. Pooled samples from the three dogs

were used for further studies. Similar, blood from a healthy canine blood donor was cultures and supernatant was processed as a negative control.

Infected host cell membrane bound B. canis *proteins*. CPDA-1 anticoagulated, leucocyte-depleted blood was washed three times with ice cold RPMI-1640 and iRBC were concentrated by a Percoll gradient (GE Healthcare) centrifugation (Figueroa et al. 1990). Parasites were then released from the iRBC by streptolysin-O mediated lysis (Richier et al. 2006) and removed by another Percoll gradient centrifugation step (Rodriguez et al. 1986). Concentrated infected RBC membranes (ghosts) were mixed with ice-cold homogenization buffer (5mM sodium phosphate, 0.1 mM EDTA, pH 8.0) and mixed vigorously, followed by repeated ultracentrifugation (40,000g for 15 min at 4 °C) until the supernatant appeared colorless and the ghosts appeared yellow-whitish. Membrane aggregates were then disrupted by mild sonication (Branson Sonifier 250, Branson Ultrasonics Corporation, 5 times 30 pulses, duty cycle 20%, output control 2.0) on ice and proteins were precipitated in chilled acetone and lipids were removed. Ghost proteins were stored in homogenization buffer at -80°C until used. The same protocol was used to collect ghost proteins from uninfected erythrocytes.

Mass spectrometry and protein identification

Different protein preparations pooled culture supernatant from blood cultures, and membrane fractions from infected and uninfected RBCs) were separated by one dimensional 10% SDS-PAGE under sterile and reducing conditions. Gels for mass spectrometry (MS) analysis were stained using Instant blue (Expedeon, Prod. ISB1L) and de-stained with sterile water.

Stained gel lanes were cut into 8 equal sections. Each section was further diced into smaller pieces and washed twice with 100 µl of 100 mM ammonium bicarbonate/ 50 % acetonitrile

for 15 min at 50 °C. The sections were dehydrated with 50 µl of acetonitrile. The gel pieces were rehydrated with 20 µl trypsin solution (5 ng/µl in 10 mM Tris-HCl/ 2 mM $CaCl_2$ at pH 8.2) and 40 µl buffer (10 mM Tris-HCl/ 2 mM $CaCl_2$ at pH 8.2). Microwave-assisted digestion was performed for 30 minutes at 60 °C with the microwave power set to 5 W (CEM Discover, CEM corp., USA). Supernatants were collected in fresh tubes and the gel pieces were extracted with 150 µl of 0.1% trifluoroacetic acid/ 50% acetonitrile. Supernatants were combined, dried, and the samples were dissolved in 20 µl 0.1% formic acid before being transferred to the autosampler vials for liquid chromatography-tandem MS (injection volume 7 to 9 µl). Samples were measured on a Q-exactive mass spectrometer (Thermo Scientific) equipped with a nanoAcquity UPLC (Waters Corporation). Peptides were trapped on a Symmetry C18, 5 µm, 180 µm x 20 mm column (Waters Corporation) and separated on a BEH300 C18, 1.7 um, 75 µm x 150 mm column (Waters Corporation) using a gradient formed between solvent A (0.1% formic acid in water) and solvent B (0.1% formic acid in acetonitrile). The gradient started at 1% solvent B and the concentration of solvent B was increased to 40% within 60 minutes. Following peptide data acquisition, different database searches were performed using the MASCOT search program against a database build on the *B. canis* annotated genes with a concatenated decoy database supplemented with commonly observed contaminants and the Swissprot database to increase database size, or against NCBI database for *Canis lupus familiaris*. The identified hits were then loaded onto the Scaffold Viewer version 4 (Proteome Software, Portland, US) and filtered based on relaxed or high stringency parameters. Relaxed stringency parameters correspond to a minimal mascot score of 20 for peptide probability, a protein probability greater than 50%, and a minimum of 1 unique peptide per protein. High stringent parameters were set to reach a corresponding false discovery rate (peptide and protein) <5% at 90% for peptide and protein probability, and 2 and 1 unique peptides per

protein for the secreted soluble and membrane-bound proteins, respectively. All datasets were corrected for obvious host- and environmental contaminants by an additional pBLAST search with the single peptide hits.

References

Aurrecoechea C, Brestelli J, Brunk BP, Fischer S, Gajria B, Gao X, Gingle A, Grant G, Harb OS, Heiges M et al. 2010. EuPathDB: a portal to eukaryotic pathogen databases. *Nucleic Acids Res* **38**: D415-419.

Bendtsen JD, Jensen LJ, Blom N, Von Heijne G, Brunak S. 2004a. Feature-based prediction of non-classical and leaderless protein secretion. *Protein Eng Des Sel* **17**: 349-356.

Bendtsen JD, Nielsen H, von Heijne G, Brunak S. 2004b. Improved prediction of signal peptides: SignalP 3.0. *J Mol Biol* **340**: 783-795.

Brameier M, Krings A, MacCallum RM. 2007. NucPred--predicting nuclear localization of proteins. *Bioinformatics* **23**: 1159-1160.

Chaisson MJ, Tesler G. 2012. Mapping single molecule sequencing reads using basic local alignment with successive refinement (BLASR): application and theory. *BMC Bioinformatics* **13**: 238.

Chin CS, Alexander DH, Marks P, Klammer AA, Drake J, Heiner C, Clum A, Copeland A, Huddleston J, Eichler EE et al. 2013. Nonhybrid, finished microbial genome assemblies from long-read SMRT sequencing data. *Nat Methods* **10**: 563-569.

Cilingir G, Broschat SL, Lau AO. 2012. ApicoAP: the first computational model for identifying apicoplast-targeted proteins in multiple species of Apicomplexa. *PLoS One* **7**: e36598.

Conesa A, Gotz S, Garcia-Gomez JM, Terol J, Talon M, Robles M. 2005. Blast2GO: a universal tool for annotation, visualization and analysis in functional genomics research. *Bioinformatics* **21**: 3674-3676.

Eid J, Fehr A, Gray J, Luong K, Lyle J, Otto G, Peluso P, Rank D, Baybayan P, Bettman B et al. 2009. Real-time DNA sequencing from single polymerase molecules. *Science* **323**: 133-138.

Emanuelsson O, Nielsen H, Brunak S, von Heijne G. 2000. Predicting subcellular localization of proteins based on their N-terminal amino acid sequence. *J Mol Biol* **300**: 1005-1016.

English AC, Richards S, Han Y, Wang M, Vee V, Qu J, Qin X, Muzny DM, Reid JG, Worley KC et al. 2012. Mind the gap: upgrading genomes with Pacific Biosciences RS long-read sequencing technology. *PLoS One* **7**: e47768.

Figueroa JV, Buening GM, Kinden DA. 1990. Purification of the erythrocytic stages of *Babesia bigemina* from cultures. *Parasitol Res* **76**: 675-680.

Finn RD, Bateman A, Clements J, Coggill P, Eberhardt RY, Eddy SR, Heger A, Hetherington K, Holm L, Mistry J et al. 2014. Pfam: the protein families database. *Nucleic Acids Res* **42**: D222-230.

Fischer S, Brunk BP, Chen F, Gao X, Harb OS, Iodice JB, Shanmugam D, Roos DS, Stoeckert CJ, Jr. 2011. Using OrthoMCL to assign proteins to OrthoMCL-DB groups or to cluster proteomes into new ortholog groups. *Curr Protoc Bioinformatics* **Chapter 6**: Unit 6 12 11-19.

Fu L, Niu B, Zhu Z, Wu S, Li W. 2012. CD-HIT: accelerated for clustering the next-generation sequencing data. *Bioinformatics* **28**: 3150-3152.

Grabherr MG, Haas BJ, Yassour M, Levin JZ, Thompson DA, Amit I, Adiconis X, Fan L, Raychowdhury R, Zeng Q et al. 2011. Full-length transcriptome assembly from RNA-Seq data without a reference genome. *Nat Biotechnol* **29**: 644-652.

Jurka J, Kapitonov VV, Pavlicek A, Klonowski P, Kohany O, Walichiewicz J. 2005. Repbase Update, a database of eukaryotic repetitive elements. *Cytogenet Genome Res* **110**: 462-467.

Korf I. 2004. Gene finding in novel genomes. *BMC Bioinformatics* **5**: 59.

Krogh A, Larsson B, von Heijne G, Sonnhammer EL. 2001. Predicting transmembrane protein topology with a hidden Markov model: application to complete genomes. *J Mol Biol* **305**: 567-580.

Kumar M, Verma R, Raghava GP. 2006. Prediction of mitochondrial proteins using support vector machine and hidden Markov model. *J Biol Chem* **281**: 5357-5363.

Li L, Stoeckert CJ, Jr., Roos DS. 2003. OrthoMCL: identification of ortholog groups for eukaryotic genomes. *Genome Res* **13**: 2178-2189.

Parra G, Bradnam K, Korf I. 2007. CEGMA: a pipeline to accurately annotate core genes in eukaryotic genomes. *Bioinformatics* **23**: 1061-1067.

Parra G, Bradnam K, Ning Z, Keane T, Korf I. 2009. Assessing the gene space in draft genomes. *Nucleic Acids Res* **37**: 289-297.

Pelle KG, Jiang RH, Mantel PY, Xiao YP, Hjelmqvist D, Gallego-Lopez GM, A OTL, Kang BH, Allred DR, Marti M. 2015. Shared elements of host-targeting pathways among apicomplexan parasites of differing lifestyles. *Cell Microbiol* **17**: 1618-1639.

Pierleoni A, Martelli PL, Casadio R. 2008. PredGPI: a GPI-anchor predictor. *BMC Bioinformatics* **9**: 392.

Quinlan AR. 2014. BEDTools: The Swiss-Army Tool for Genome Feature Analysis. *Curr Protoc Bioinformatics* **47**: 11 12 11-11 12 34.

Reid AJ, Blake DP, Ansari HR, Billington K, Browne HP, Bryant J, Dunn M, Hung SS, Kawahara F, Miranda-Saavedra D et al. 2014. Genomic analysis of the causative agents of coccidiosis in domestic chickens. *Genome Res* **24**: 1676-1685.

Richier E, Biagini GA, Wein S, Boudou F, Bray PG, Ward SA, Precigout E, Calas M, Dubremetz JF, Vial HJ. 2006. Potent antihematozoan activity of novel bisthiazolium drug T16: evidence for inhibition of phosphatidylcholine metabolism in erythrocytes infected with *Babesia* and *Plasmodium* spp. *Antimicrob Agents Chemother* **50**: 3381-3388.

Rodriguez SD, Buening GM, Vega CA, Carson CA. 1986. *Babesia bovis*: purification and concentration of merozoites and infected bovine erythrocytes. *Exp Parasitol* **61**: 236-243.

Sriprawat K, Kaewpongsri S, Suwanarusk R, Leimanis ML, Lek-Uthai U, Phyo AP, Snounou G, Russell B, Renia L, Nosten F. 2009. Effective and cheap removal of leukocytes and platelets from *Plasmodium vivax* infected blood. *Malar J* **8**: 115.

Stanke M, Waack S. 2003. Gene prediction with a hidden Markov model and a new intron submodel. *Bioinformatics* **19 Suppl 2**: ii215-225.

Supplemental Figure S1. Workflow and criteria for the iprediction of the *Babesia canis* exportome. Simplified and schematic representation of exportome parsing. cSP: canonical signal peptid; TMD: transmembrane domain; GPI: predicted glycophosphatidylinositol; a/nc SPW: alternative/non classical secretion pathway.

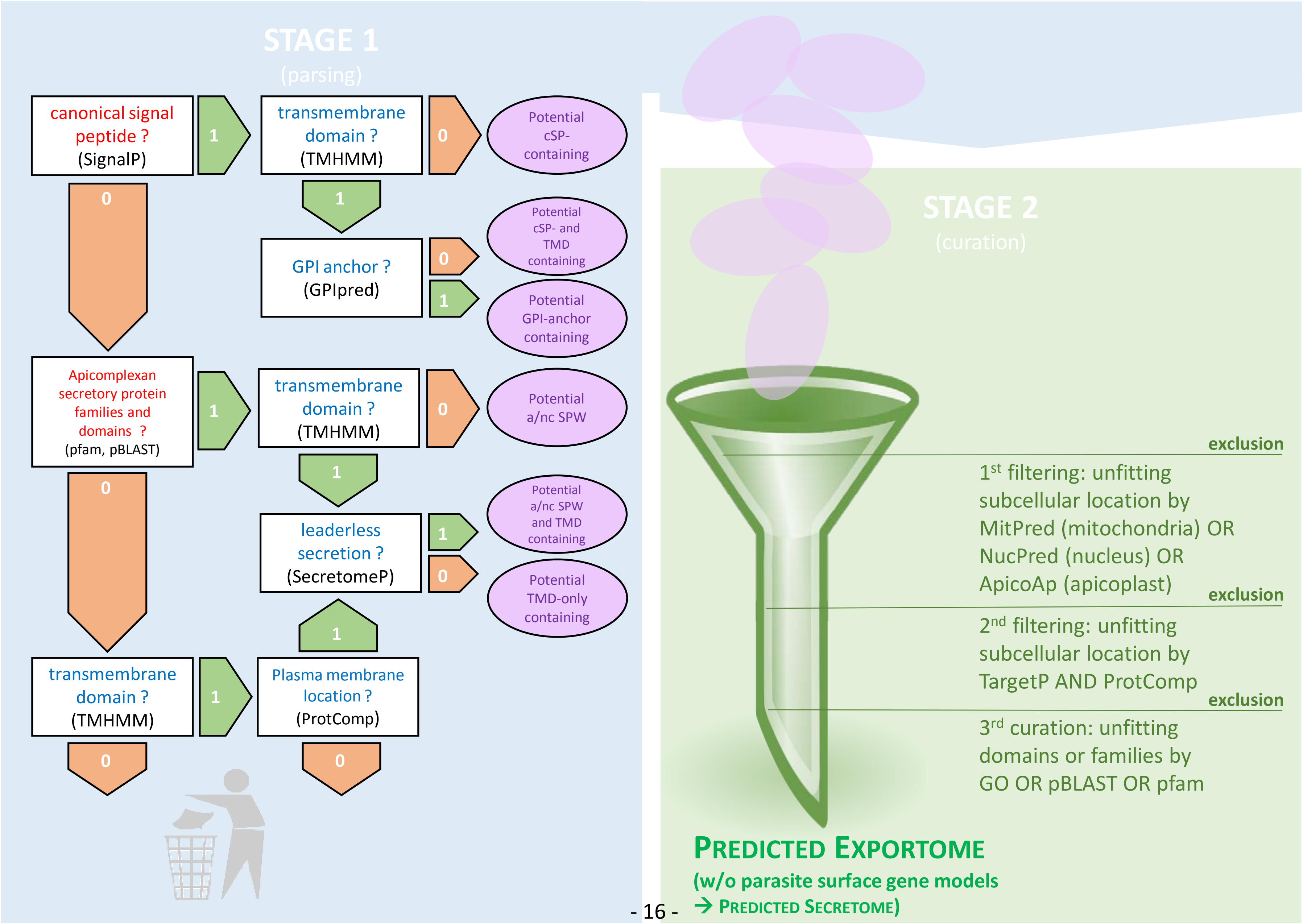

Supplemental Figure S2. Specific *B. canis* exported proteins. (A) Venn diagram of *B. canis* specific proteins detected in short-term culture supernatants of infected erythrocytes compared to culture supernatant from uninfected erythrocytes, and (B) infected erythrocyte membranes compared with uninfected erythrocytes. (C) Comparison of the two dataset reveal the predicted set of *B. canis* exported and host interacting factors. *Bc*: *Babesia canis*; iEc: infected erythrocytes; cRBC: canine red blood cell

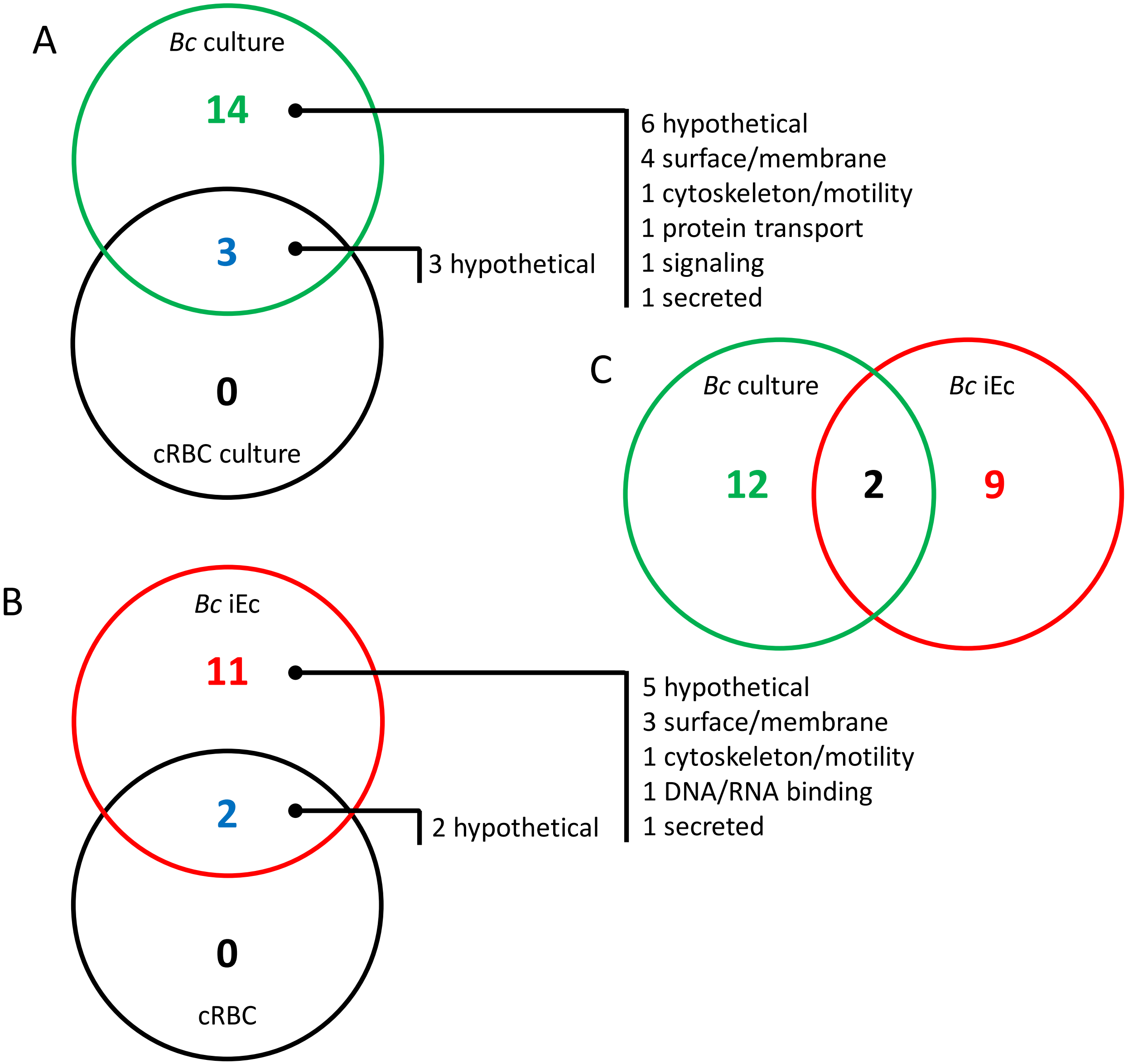

Supplemental Figure S3. Parasitaemia, prognostic markers (related to severity of disease), and acute phase response (by cCRP) in three dogs (solid line, broken line, and dotted line, respectively) experimentally infected with *B. canis*. The graph was normalized to the very first signs of acute crisis detected by any clinical signs of acute shock or central nervous depression, which was 126, 145 and 147 hours post inoculation, respectively. The shaded grey areas represent reference intervals. The hatched area correspond to the period of clinical deterioration. WBC: white blood cells; cCRP: canine C-reactive protein.

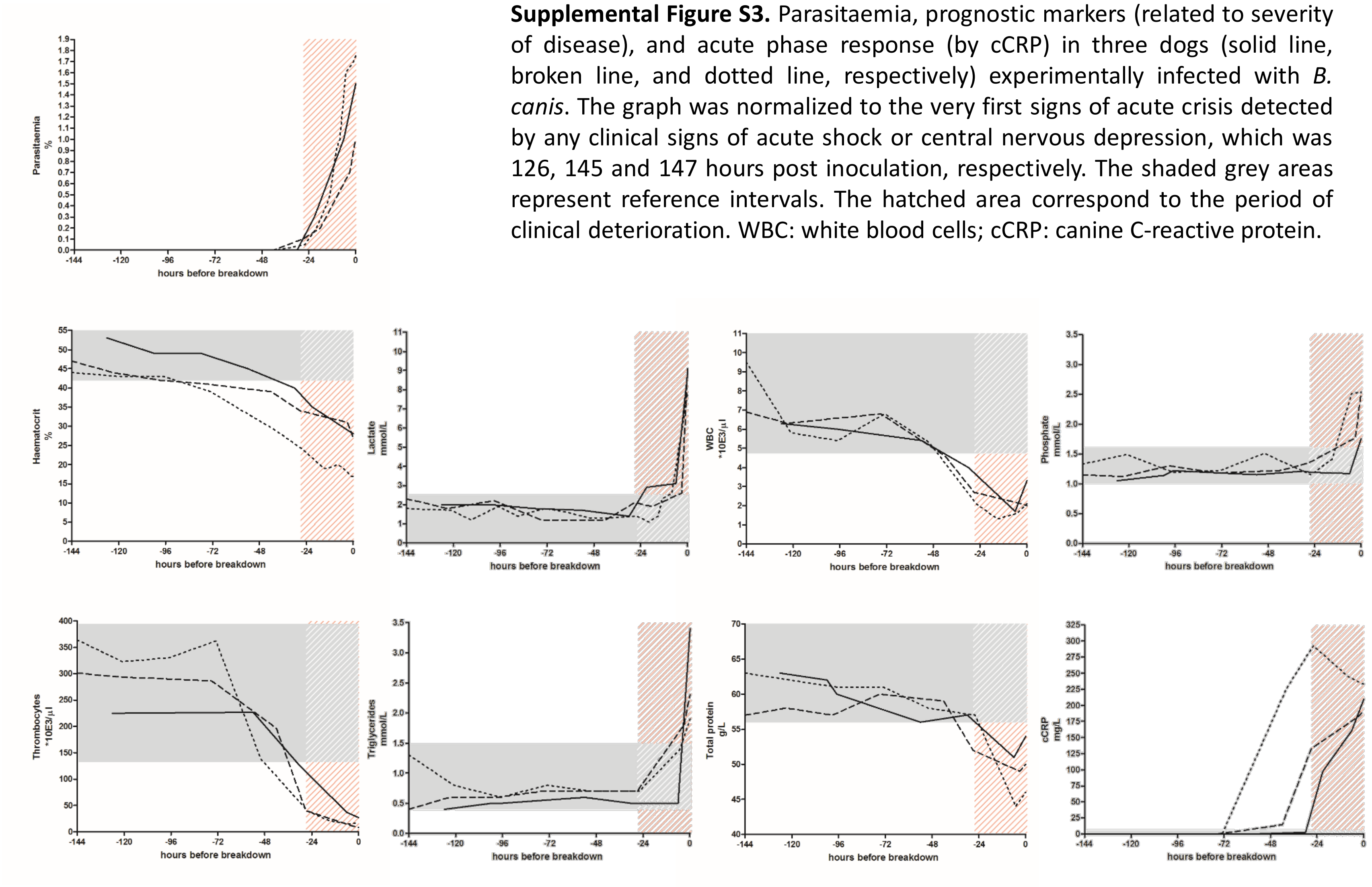

Supplement: Supplementary file 1 — Supplementary information [file 41598_2017_3445_MOESM1_ESM.pdf]
